# Supplementary material for: The Buffering Effect of Awe on Negative Emotions in Self-Threatening Situations
Source: Behav Sci (Basel). 2023 Jan 4;13(1):44. doi: 10.3390/bs13010044 (PMC9854425; doi:10.3390/bs13010044)
Supplement: Supplementary file 1 [file behavsci-13-00044-s001.zip › behavsci-2035214-supplementary.pdf]

Supplementary Materials

1. Research framework

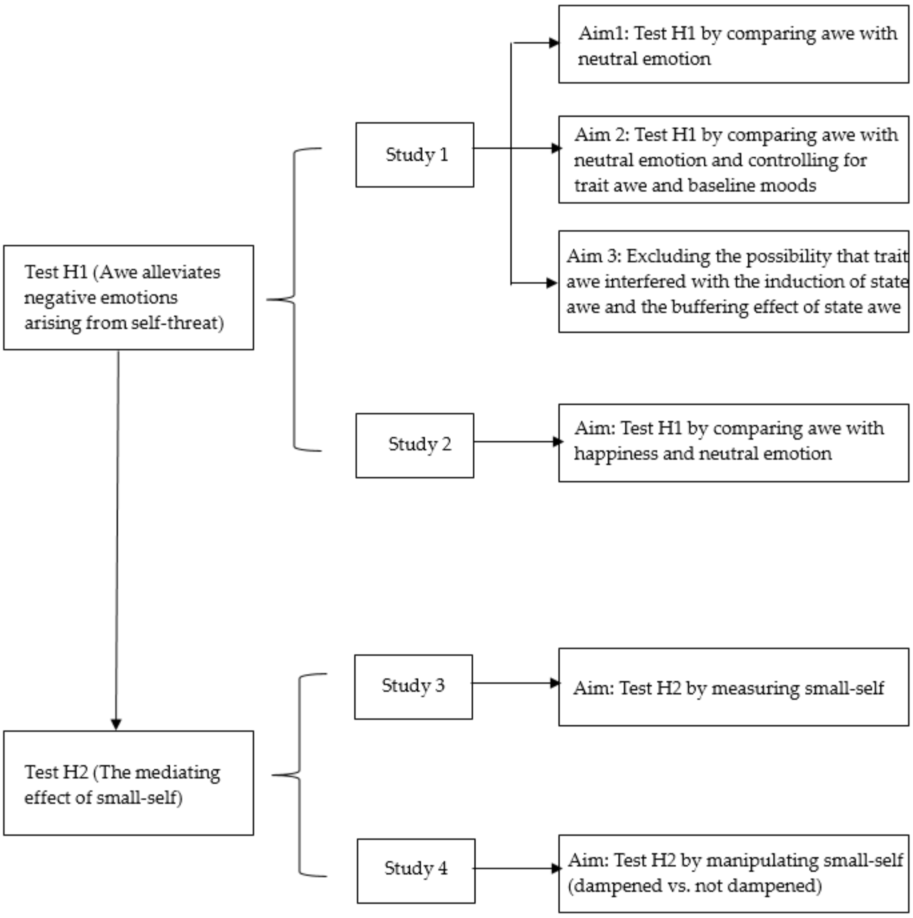

Figure S1. Research framework.

2. The steps of each study

Table S1. The steps of each study.

Study	Step 1	Step 2	Step 3	Step 4	Step 5	Step 6
Study 1	assessed trait awe and baseline mood	induced intelligence-threat	induced emotion (awe vs. neutral)	assessed negative emotions arising from self-threat	conducted the manipulation check of awe (vs. neutral)	collected demographics information

Study 2	induced appearance-threat	induced emotion (awe vs. neutral vs. happiness)	assessed negative emotions arising from self-threat	conducted the manipulation check of awe (vs. neutral vs. happiness)	collected demographics information	/
Study 3	induced appearance-threat	induced emotion (awe vs. neutral)	assessed negative emotions arising from self-threat	assessed small-self	conducted the manipulation check of awe (vs. neutral)	collected demographics information
Study 4	induced power-threat	induced emotion (awe vs. neutral)	manipulated small-self	assessed negative emotions arising from self-threat	conducted the manipulation check of awe (vs. neutral)	collected demographics information

3. Video links

Neutral video S1：https://www.bilibili.com/video/BV1A64y1U7vg?share_source=copy_web

Neutral video S2：https://www.bilibili.com/video/BV1wy4y147yf?share_source=copy_web

Awe video S1：https://www.bilibili.com/video/BV16Z4y1c7c5?share_source=copy_web

Awe video S2：https://www.bilibili.com/video/BV1x84y1F7nk?share_source=copy_web

Happy video：https://www.bilibili.com/video/BV1R64y1m73Z?share_source=copy_web

4. Statistical details of studies

Table S2. Statistical details of studies.

Study		Study 1		Study 2	Study 3		Study 4
Participants		151		180	147		252
Design		2 (emotion induction: awe vs. neutral)		3 (emotion induction: awe vs. happiness vs. neutral)	2 (emotion induction: awe vs. neutral)		2 (emotion induction: awe vs. neutral) * 2 (small-self: dampened vs. not dampened)
Demographics		Mage = 27.13, SDage = 6.28,<br>54 females		Mage = 27.60, SDage = 6.52,<br>87 females	Mage = 28.52, SDage = 7.00,<br>70 females		Mage = 27.74, SDage = 5.53,<br>124 females
Aim		Test H1 (awe vs. neutral emotion)		Test H1 (awe vs. happiness vs. neutral emotion)	Test H2 (measure small-self)		Test H2 (manipulate small-self)
Manipulation check	Method	ANOVA		as in Study1	as in Study 1		as in Study 1
	DV	condition (awe = 1, neutral = 0)		condition (awe = 1, neutral = 0, happiness = 2)	as in Study 1		as in Study 1
	IV	sad, fear, quiet, awe, pride, happy, and excited		as in Study1	as in Study 1		as in Study 1
	Covariates	gender, age, and educational background.		as in Study1	as in Study 1		as in Study 1
	Results	awe: Mawe = 5.88, SDawe = 1.37; Mneu = 3.04, SDneu = 1.36; F (1, 146) = 155.69, p < .01, η2 = .52<br>there were a few differences in pride (F (1, 146) = 4.39, p = .04, η2 = .03), fear (F (1, 146) = 4.14, p = .04, η2 =.03), and quiet (F (1, 146) = 6.13, p = .01, η2 = .04) between the two conditions<br>No differences in the other emotions between the two conditions were found.		Details in verses 246-256	awe: Mawe = 5.55, SDawe = 1.52; Mneu = 2.42, SDneu = 1.47; F (1,142) = 150.53, p < .01, η2 = .52<br>The two conditions had no significant difference in other emotions.		awe: Mawe = 5.30, SDawe = 1.87; Mneu = 3.53, SDneu = 1.93; F (1, 247) = 54.42, p < .01, η2 = .18<br>The two conditions had no significant difference in other emotions.
Hypothesis test	Method	ANOVA	ANOVA	ANOVA	ANOVA (main effect)	Process-Model 4 (mediating effect)	two-way ANOVA
	DV	condition (awe = 1, neutral = 0)	condition (awe = 1, neutral = 0)	condition (awe = 1, control = 0, happiness = 2)	as in Study 1	condition (awe = 1, neutral = 0)	condition (awe = 1, neutral = 0)<br>small-self (dampened = 1, not dampened = 0)
	IV	Negative emotions arising from self-threat	Negative emotions arising from self-threat	Negative emotions arising from self-threat	Negative emotions arising from self-threat	Negative emotions arising from self-threat	Negative emotions arising from self-threat
	Mediator	/	/	/	/	small-self	/
	Covariates	pride, fear, and quiet	pride, fear, quiet, trait awe, baseline moods,age, gender, and educational background	age, gender, educational background, excited,	age, gender, and educational background	age, gender, and educational background	age, gender, and educational background
	Results	Mawe = 2.02, SDawe = 1.27; Mneu = 3.46, SDneu = 1.57; F (1, 146) = 26.02, p < .01, η2 = .15	Mawe = 2.02, SDawe = 1.27; Mneu = 3.46, SDneu = 1.57; F (1, 137) = 26.28, p < .01, η2 = .16	**awe vs. neutral vs. happiness**: F (2, 174) = 10.93, p < .01, η2 = .11<br>**awe vs. neutral**: Mawe = 3.08, SDawe = 1.19; Mneu = 3.90, SDneu = 1.29; F (1, 113) = 9.79, p < .01, η2 =.08<br>**awe vs. happiness**: Mawe = 3.08, SDawe = 1.19; Mhap = 3.96, SDhap = 1.35; F (1, 115) = 14.69, p < .01, η2 = .11<br>**neutral vs. happiness**: Mneu = 3.90, SDneu = 1.29; Mhap = 3.96, SDhap = 1.35; F (1, 114) = 3.38, p = .07, η2 = .03	Mawe = 2.71, SDawe = 1.32; Mneu = 3.67, SDneu = 1.65; F (1, 142) = 14.35, p < .01, η2 = .09	**awe - small-self**: b = .58, se = .07, t = 8.59, p < .001<br>**small-self - negative emotions**: b = -.33, se = .10, t = -3.42, p < .01<br>**mediating effect**: b = -.19, 95% CI [-.35, -.06]	**interaction effect**: F (1, 245) = 5.59, p = .02, η2 = .02<br>specific effect:<br>**in the small-self not-dampened condition**: Mawe = 2.36, SDawe = 1.55; Mneu = 3.61, SDneu = 1.84; F (1, 245) = 16.08, p < .01, η2 = .07<br>**in the small-self dampened condition**: Mawe = 3.13, SDawe = 1.73; Mneu = 3.30, SDneu = 1.81; F (1, 245) = .42, p = .52, η2 = .001

5. The results of pre-study

Self-Perception after Self-Threat. The result from the one-way analysis of variance (ANOVA) showed that participants perceived less appearance attractiveness in the appearance-threat (*M* = 3.02, *SD* = 1.29) than neutral group (*M* = 5.24, *SD* = 1.20), *F* (4, 92) = 75.20, $p < .001$, $\eta^2 = .45$. Participants perceived less power in power-threat (*M* = 2.15, *SD* = 2.20) than neutral group (*M* = 5.06, *SD* = 1.56), *F* (4, 122) =127.06, $p < .001$, $\eta^2 = .51$. Participants perceived less intelligence in intelligence-threat (M = 3.29, SD = 1.26) than neutral group (*M* = 4.77, *SD* = 1.30), *F* (4, 131) = 45.52, $p < .001$, $\eta^2 = .26$.

Negative Emotion towards Self-Threat. Participants experiencing appearance-threat reported higher negative emotion (*M* = 4.25, *SD* = 1.57) than controls (*M* = 2.70, *SD* = .96), *F* (4, 92) = 31.84, $p < .001$, $\eta^2 = .26$. Participants experiencing power-threat reported higher negative emotion (*M* = 4.57, *SD* = 1.58) than controls (*M* = 2.36, *SD* = 1.36), *F* (4, 122) = 67.35, $p < .001$, $\eta^2 = .36$. Participants experiencing intelligence threat reported higher negative emotion (*M* = 3.69, *SD* = 1.67) than controls (*M* = 2.46, *SD* = 1.26), *F* (4, 131) = 22.98, $p < .001$, $\eta^2 = .15$.

Correlation. The Pearson correlation analysis showed that the correlation between negative emotion towards self-threat and appearance-perception ($r = -.76$, $p < .001$), power ($r = -.80$, $p < .001$) and intelligence ($r = -.71$, $p < .001$) were negative and strong.
